# Early intervention for trauma and loss: overview and working care model

**DOI:** 10.3402/ejpt.v6.28543

**Published:** 2015-06-11

**Authors:** Brett Litz

**Affiliations:** 1 Massachusetts Veterans Epidemiology Research and Information Center (MAVERIC), VA Boston Healthcare System, Boston, MA, USA; 2 Department of Psychology, College of Arts and Sciences, Boston University, Boston, MA, USA; 3 Department of Psychiatry, Boston University School of Medicine, Boston, MA, USA

There is a lack of clarity and consensus in the applied and research communities about the methods and goals for early intervention in the context of exposure to potentially traumatizing events (PTEs; Litz & Gray, 2004). Since 9/11, the research community has tended to see prevention in a medical model frame, namely as early interventions designed to prevent new-onset or relapsed mental disorders (and serious disruptions in the functional capacities). In this context, researchers have rightfully eschewed approaches that assume that all people exposed to PTEs are at equal risk for stress disorder and prolonged grief, or disruptions in functioning, and require some kind of preventative intervention, typically provided in an intrusive, too-early, and chiefly one-off manner (e.g., grief counseling, debriefing; Gray, Litz, & Papa, 2006; Litz, Gray, Bryant, & Adler, 2002).

There is indeed good evidence to suggest that most people exposed to PTEs will not develop posttraumatic stress disorder (PTSD) or other serious mental and behavioral disorders (Bonanno, 2004). But there is a lack of consensus about how to operationalize resilience in the face of PTEs (Litz, Steenkamp, et al., 2014). Resilience is important to study well because it can provide clues about what happens to people without formal intervention as well as data about the characteristics of individuals, contexts, cultures, and communities that confer risk or serve as protective factors. Many see resilience as a lack of symptoms or impairment (e.g., Bonanno, 2004; Southwick et al., 2014). Yet, the research used to support this supposition has confounded degree and type of exposure to PTEs, and the response to the exposure. Researchers tend to overgeneralize from large-scale studies of reactions to broad PTEs, such as the 9/11 attacks on residents of New York City. In these studies, consistently, apparent resilience, i.e., low symptom trajectories, is chiefly the result of peripheral exposure to PTEs (Litz, Steenkamp, et al., 2014; Nash et al., 2015; Steenkamp, Dickstein, Salters-Pedneault, Hofmann, & Litz, 2012). When exposure to PTEs is frequent and proximal, or when the PTEs entail human maliciousness or exposure to grotesque violence, the expectation of non-response is unsupported, and unsurprisingly so.

In this presentation, I will review and define the concept of resilience and share research studies that have confirmed the expectation that exposure and context matter. Unfortunately, the expectation of normatively low symptoms and impairment has led to an overgeneralized “let people be” mind-set clinically. This is valid in some contexts but not others. Unexposed family members, leaders, and community members, for some PTEs, and in some contexts and cultures, would legitimately feel abandoned by the care community if helping professionals disengaged, waiting for salient signs of psychopathology to be manifest in only the few. One of the important questions for the field is what should we do to respond to the work systems, family, and community needs and how should this be framed? In this presentation, I will critically review the relatively recent history of varied models of early intervention for PTEs and I will provide an overview of the science and practical application of prevention strategies in the aftermath of exposure to PTEs (Litz & Maguen, 2007; Litz & Bryant, 2009). I will also offer a conceptual scheme to understand and contextualize prevention approaches that is more useful than the relatively outdated “primary,” “secondary,” and “tertiary” prevention framework (Muñoz, Mrazek, & Haggerty, 1996). I will also provide several examples of successful and unsuccessful approaches to prevention in the context of the US military (Steenkamp, Nash, & Litz, 2013) and a study of bereaved caregivers at risk for prolonged grief disorder (Litz, Schorr, et al., 2014). I will end by describing the challenges that lie ahead for researchers, decision makers, and care providers, and I will provide a checklist of issues that need to be problem-solved when planning early intervention strategies in the field.

This conference was funded by a grant from The Swedish Foundation for Humanities and Social Sciences (F14-1747:1).
